# Technology-Delivered Adaptations of Motivational Interviewing for the Prevention and Management of Chronic Diseases: Scoping Review

**DOI:** 10.2196/35283

**Published:** 2022-08-09

**Authors:** Havisha Pedamallu, Matthew J Ehrhardt, Julia Maki, April Idalski Carcone, Melissa M Hudson, Erika A Waters

**Affiliations:** 1 Division of Public Health Sciences Department of Surgery Washington University in St Louis St Louis, MO United States; 2 Department of Oncology, Department of Epidemiology and Cancer Control St. Jude Children’s Research Hospital Memphis, TN United States; 3 Department of Family Medicine and Public Health Sciences Wayne State University School of Medicine Detroit, MI United States; 4 Division of Public Health Sciences Department of Surgery Washington University in St Louis, School of Medicine St Louis, MO United States

**Keywords:** motivational interviewing, technology, telehealth, health behavior, chronic disease, socioeconomic factors, health promotion, disease management, primary prevention, secondary prevention, minority health

## Abstract

**Background:**

Motivational interviewing (MI) can increase health-promoting behaviors and decrease health-damaging behaviors. However, MI is often resource intensive, precluding its use with people with limited financial or time resources. Mobile health–based versions of MI interventions or technology-delivered adaptations of MI (TAMIs) might increase reach.

**Objective:**

We aimed to understand the characteristics of existing TAMIs. We were particularly interested in the inclusion of people from marginalized sociodemographic groups, whether the TAMI addressed sociocontextual factors, and how behavioral and health outcomes were reported.

**Methods:**

We employed the PRISMA (Preferred Reporting Items for Systematic Reviews and Meta-Analyses) guidelines for scoping reviews to conduct our scoping review. We searched PubMed, CINAHL, and PsycInfo from January 1, 1996, to April 6, 2022, to identify studies that described interventions incorporating MI into a mobile or electronic health platform. For inclusion, the study was required to (1) describe methods/outcomes of an MI intervention, (2) feature an intervention delivered automatically via a mobile or electronic health platform, and (3) report a behavioral or health outcome. The exclusion criteria were (1) publication in a language other than English and (2) description of only in-person intervention delivery (ie, no TAMI). We charted results using Excel (Microsoft Corp).

**Results:**

Thirty-four studies reported the use of TAMIs. Sample sizes ranged from 10 to 2069 participants aged 13 to 70 years. Most studies (n=27) directed interventions toward individuals engaging in behaviors that increased chronic disease risk. Most studies (n=22) oversampled individuals from marginalized sociodemographic groups, but few (n=3) were designed specifically with marginalized groups in mind. TAMIs used text messaging (n=8), web-based intervention (n=22), app + text messaging (n=1), and web-based intervention + text messaging (n=3) as delivery platforms. Of the 34 studies, 30 (88%) were randomized controlled trials reporting behavioral and health-related outcomes, 23 of which reported statistically significant improvements in targeted behaviors with TAMI use. TAMIs improved targeted health behaviors in the remaining 4 studies. Moreover, 11 (32%) studies assessed TAMI feasibility, acceptability, or satisfaction, and all rated TAMIs highly in this regard. Among 20 studies with a disproportionately high number of people from marginalized racial or ethnic groups compared with the general US population, 16 (80%) reported increased engagement in health behaviors or better health outcomes. However, no TAMIs included elements that addressed sociocontextual influences on behavior or health outcomes.

**Conclusions:**

Our findings suggest that TAMIs may improve some health promotion and disease management behaviors. However, few TAMIs were designed specifically for people from marginalized sociodemographic groups, and none included elements to help address sociocontextual challenges. Research is needed to determine how TAMIs affect individual health outcomes and how to incorporate elements that address sociocontextual factors, and to identify the best practices for implementing TAMIs into clinical practice.

## Introduction

### Background

Chronic diseases, such as heart disease, cancer, and diabetes, are the leading causes of death in the United States, affecting 6 in 10 adults [1]. The risk of developing many chronic diseases and their corresponding complications, if diagnosed, can be reduced by avoiding the following 4 key health behaviors: tobacco use, poor nutrition, physical inactivity, and excessive alcohol use [1]. However, reducing these harmful behaviors can be exceptionally difficult. As a result, a multitude of interventions have been developed that attempt to facilitate health behavior change in these domains [2].

### Using eHealth Technologies to Improve Health

The use of computing and internet technologies generally (eHealth), and smartphone and texting technologies specifically (mobile health [mHealth]), in health behavior change interventions has greatly increased over the past 3 decades [3]. These technologies can be effective tools for delivering health behavior interventions to diverse populations for a variety of behavior change goals. To illustrate, a systematic review of mobile apps and text messaging interventions demonstrated improvement across a variety of physical and mental health outcomes, including weight loss, smoking cessation, medication adherence, and depression and anxiety symptoms [4]. Similar findings were reported in a systematic review of text messaging health promotion interventions [5]. mHealth interventions may also be useful for facilitating self-management in patients with chronic diseases, such as improving medication adherence and control of chronic disease indicators like BMI, and activating and empowering patients [4-7].

mHealth technologies in particular have the potential to greatly improve health care access, improve delivery processes, and reduce chronic care costs [6], especially in areas that are medically underserved and underresourced, and in areas where internet access is limited to personal mobile phones [8]. mHealth technologies are widely accessible, popular across sociodemographic groups, and portable, and can facilitate timely interventions for patients [8,9]. These features are especially important for encouraging chronic disease prevention and management behaviors, which require timely and frequent reminders and interactions with patients that are impractical for health care practitioners to provide in office settings.

The accessibility of mHealth technologies may be especially important for people from sociodemographic groups that have been underserved, mistreated, or marginalized by biomedical research and practice (hereafter *marginalized*). For example, patients who live in rural areas face long travel and wait times, which may limit how often and for how long they can meet with their providers [10]. People from marginalized racial or ethnic groups face additional barriers, including medical distrust, which stems in large part from experiencing stigma, discrimination, and racism from health care systems and providers [11].

The effectiveness of eHealth and mHealth technologies for health promotion and management behaviors, combined with their availability and accessibility to the public, suggests that these platforms are effective tools for increasing the reach of interventions to greater numbers of individuals with sociocontextual challenges. However, the potential benefits of technology-delivered interventions may be offset by the fact that standard eHealth and mHealth technologies might not motivate patients in a way that fosters autonomy, which is critical to maintaining behavior change over time [12].

### Motivational Interviewing and Its Use in eHealth Interventions

Motivational interviewing (MI) is a method of talking to people about changing their behavior [13]. The goal of MI is to increase intrinsic motivation and self-efficacy for engaging in health promoting behaviors using patient-centered yet directive communication techniques [14]. Specifically, MI counselors rely on reflexive listening, strategic questions, affirmations of character strengths, and statements emphasizing patients’ decision-making autonomy to elicit “change talk” [15]. Change talk involves statements expressing patients’ own desires, abilities, reasons, needs, and commitments to change their behavior while embodying “MI spirit,” an empathetic, collaborative, and nonjudgmental demeanor. There is strong evidence supporting MI as a strategy to address barriers to effective health behavior counseling [16]. While health care professionals may attempt to persuade patients to adhere to recommended health behaviors, MI encourages personal decision-making about change and provides guidance and support about potential mechanisms of change [17,18]. During an MI session, a counselor uses the principles of the self-actualization theory and free choice to help individuals identify and work toward their goals​ [19,20]. MI can include health education and address sociocontextual factors that constrain an individual’s choices. MI has been effective in promoting health behavior change for individuals with and without chronic diseases [21,22]. However, while MI offers many benefits to patients, its reach may be limited as employing trained counselors is expensive and training other health care providers may be time consuming and resource intensive. 

Technology-delivered adaptations of MI (TAMIs) have been developed to combine the useful features of MI interventions (eg, promoting patient autonomy) with the benefits of mHealth interventions (eg, increase accessibility while limiting costs to patients and the health care system). Despite the complexity of developing TAMIs for intervention studies seeking to improve health behaviors, a 2016 systematic review reported that they are feasible to implement and well accepted by patients [23].

### Objective and Research Questions

The objective of this research was to gain an understanding of the characteristics and outcomes of TAMIs that were not addressed in a previous review [23]. Specifically, we asked the following questions: (1) To what extent do TAMIs include individuals from marginalized sociodemographic groups? (2) How do TAMIs address sociocontextual influences on health (eg, the built environment and financial barriers)? (3) How do studies that report TAMIs describe their effects on behavioral and health outcomes?

This work is important for several reasons. First, the answers to the first 2 research questions (ie, including individuals from marginalized sociodemographic groups and addressing sociocontextual influences on health) have not been previously addressed, yet they are critical for expanding access of these potentially useful interventions to individuals and groups who have been socially marginalized or systematically and intentionally excluded from or underrepresented in biomedical research. Second, there have been many technological advancements made over the past 6 years that might shape the nature and effectiveness of TAMIs (eg, increased sophistication of tailoring interactions to participant responses) [24]. Although the previous review evaluated the effects of TAMIs on behavioral and psychological outcomes [23], examination of more modern interventions is informative considering rapidly emerging novel technologies.

We sought to map key concepts and knowledge gaps about TAMIs, including identifying the number of studies that include participants from underrepresented populations and the number of studies that address sociocontextual factors. Because these goals are more consistent with the goals of a scoping review than a systematic review [25], we conducted a scoping review.

## Methods

We employed the PRISMA (Preferred Reporting Items for Systematic Reviews and Meta-Analyses) guidelines for scoping reviews [26,27]. We did not preregister the review protocol.

JM and HP searched the PubMed, CINAHL, and PsycInfo databases for articles published from January 1, 1996, through April 6, 2022, that met the following inclusion criteria: (1) the publication described conducting an MI intervention, (2) the intervention was incorporated into an automated mobile or electronic health platform, and (3) the article reported behavioral or health outcomes.

The exclusion criteria were as follows: (1) the article was published in a language other than English and (2) the MI intervention was administered only in-person (ie, no mHealth or eHealth element).

We used the following search string to identify potentially eligible articles: ((“mHealth” OR (“m-Health”)) OR ((“text message”) OR (“text-message”) OR (“text messaging”) OR (“text-messaging”) OR (“ehealth”) OR (“e-health”) OR (“web-based”) OR (“electronic health”) OR (“technology-based”)) AND ((“motivational interviewing”) OR (“motivational interview”) OR (“intrinsic motivation”)) AND (“intervention”).

We reviewed the reference lists of identified articles and authors’ files for additional studies missed by the search criteria. HP reviewed abstracts and full-length articles. HP last searched the literature for articles to include in this review on April 6, 2022.

HP used Microsoft Excel (Microsoft Corp) to abstract the following data for each study: type of data collected (quantitative, qualitative, or mixed), study design, theoretical or conceptual model, study population, intervention target, eligibility criteria, intervention description, mHealth tool details, measures, sample size, sample characteristics, results, conclusions, and key limitations. Not all articles reported all data elements; we noted such instances as “NR” (not reported). The rows in the spreadsheet were individual articles, and the columns were individual data elements.

We have summarized the data using tables (Multimedia Appendix 1 and Multimedia Appendix 2) that are separated by the age category of participants (ie, younger than 18 years of age vs 18 years or older) and by the type of outcome (ie, behavioral vs health outcome). We have also described in the text the number and percentage of articles that had different methodological characteristics.

## Results

### Overview

**Figure 1:**
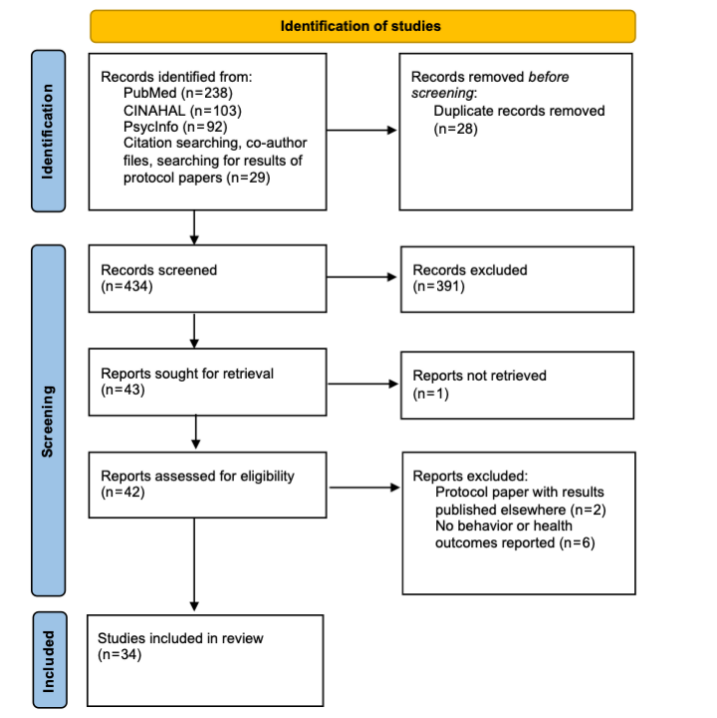
PRISMA (Preferred Reporting Items for Systematic Reviews and Meta-Analyses) flow diagram [27] for the scoping review.

We identified 34 studies reporting unique TAMIs (Figure 1). Most were conducted in the United States (n=22) [28-49]. Others were conducted in the Netherlands (n=4) [50-53], Switzerland (n=2) [54,55], Germany (n=1) [56], Korea (n=1) [57], Austria (n=1) [58], Canada (n=1) [59], and New Zealand (n=1) [60]. One study included participants from Germany, Sweden, Belgium, and the Czech Republic [61]. Detailed information about each study’s design, population, and outcomes is provided in Multimedia Appendix 1.

### General Study Information

#### Conceptual Framework

Studies were based on a variety of conceptual frameworks. Of the 34 studies, 24 used only MI [28-32,34,35,37-39,​^41^-^44^,^46^,^47^,^49^,^51^,^53^,^54^,^56^,^59^-^61^] and the remaining 10 used alternative frameworks in conjunction with MI [33,36,40,45,48,50,52,55,57,58] (Multimedia Appendix 1). The most commonly used conceptual framework other than MI was cognitive behavioral therapy (CBT; n=3) [45,50,55].

#### Study Design

The studies used a variety of designs. There were 30 randomized controlled trials (RCTs), 3 studies that used a nonrandomized pretest-posttest design with no control group [33,36,59], and 1 randomized experiment whose purpose was to refine the contents of the TAMI [58] (Multimedia Appendix 1). Control groups included active control (n=14) [29,31,34,35,37,​^39^-^41^,^43^,^45^,^47^,^53^,^54^,^57^], treatment as usual (n=7) [28,30,42,44,48,49,60], wait list (n=5) [32,50,52,55,56], or no treatment (n=4) [38,46,51,61].

#### Study Population

The study populations among the selected studies varied widely (Multimedia Appendix 1). Studies ranged in size from 10 to 2069 participants (mean 341, median 136). Six studies included participants between 13 and 18 years of age. One study recruited from the general population [51], and 2 specifically recruited individuals with mental health diagnoses [31,32]. The 31 remaining studies focused on individuals who were engaging in behaviors that increased their risk of either developing new or exacerbating existing chronic health conditions.

#### Purpose

The purpose of the health behavior interventions fell into 2 broad categories (Multimedia Appendix 1). Twenty-seven interventions were designed to prevent disease among individuals in the general population or among those engaging in behaviors that put them at a higher risk of being diagnosed with a chronic disease [29-31,33,34,36,38-40,42-48,​^50^-^57^,^59^-^61^]. The remaining 7 interventions were for encouraging chronic disease management [28,32,35,37,41,49,58].

#### Behaviors and Health Outcomes

Targeted health behaviors included the following: substance, alcohol, or tobacco use (n=24) [29-32,34,36,38-40,42-44,​^46^-^48^,^50^,^53^-^57^,^59^-^61^]; diet or physical activity (n=2) [51,52]; treatment and medication adherence (n=4) [28,35,37,41]; mental health (n=1) [45]; and risky sexual behaviors (n=2) [33,47] (Multimedia Appendix 1). One study addressed both substance, alcohol, or tobacco use and risky sexual behaviors [47]. The most common chronic disease targeted for self-management interventions was diabetes (n=2) [28,35]. Targeted health outcomes included control of diabetes (n=2) [28,35], reduction of HIV viral load (n=1) [41], reduction in depressive symptoms (n=1) [45], reduction in BMI (n=1) [49], improvement in asthma symptoms (n=1) [37], and reduction in anorexia symptoms (n=1) [58].

#### Delivery

Most studies relied on text messaging or web-based tools to deliver their intervention (Multimedia Appendix 1). Eight used automated text messaging [28,33,38-40,46,49,60], 22 used a web-based intervention [30-32,34,35,41-44,47,48,50-59,61], 1 used an app combined with automated text messaging [36], and 3 used a web-based intervention and automated text messaging [29,37,45]. None used human-generated or chat bot–generated text messaging. Of the 25 studies that used a web-based intervention or a web-based intervention + text messaging, 8 used the Computerized Intervention Authorizing Software (CIAS; [62]) [29,30,34,35,37,41,42,47]. Studies with long-term engagement often used text message–based interventions.

CIAS is a mobile or web-based platform designed to create and launch behavioral health interventions, including those based on MI. CIAS includes an avatar that is capable of over 50 animated expressions and guides participants through questions, allowing the TAMI to more accurately mimic one-on-one conversations [29]. All 8 studies that used CIAS and 3 other studies included an avatar that interacted with participants and delivered the intervention [29,30,34,35,37,41-43,47,51,53]. In 1 study that included an avatar but did not use CIAS [51], participants could select either a male or female avatar whose appearance was designed based on focus group interviews asking the target population about how they believed a motivating and reliable avatar would look. The avatar communicated with speech movements and text displayed in balloons, and featured a limited number of nonverbal expressions. Although the avatar + intervention group did not show a significant improvement in the target outcome compared to the content-identical intervention without the avatar, the authors noted that the avatar had limited relational skills that may have precluded the development of a strong relationship with the user.

### Inclusion of Individuals From Marginalized Populations

There was considerable variation in the extent to which studies included members of populations that have been marginalized (Multimedia Appendix 1). Among the 34 studies, 2 interventions were specifically designed for use by African American young people [35,37]. Moreover, 22 studies had samples that included an overrepresentation of people from marginalized sociodemographic groups in the general US population. Specifically, 14 studies recruited ≥22% African American participants [29,31,32,35,37-39,41-43,46-49], whereas African Americans represent 13.4% of the US population [63]. Five studies included ≥30% Hispanic/Latino participants [28,29,33,44,47], whereas 18.5% of the US population identifies as Hispanic/Latino [63]. One study reported that 50% of its participants identified with racial or ethnic groups other than white [34]. Three studies reported at least 67% of participants as having limited incomes [42,43,45], although TAMIs were not specifically designed for use by these populations. The remaining 14 studies had samples that either included fewer people from marginalized racial or ethnic groups than was representative in the US population or did not provide participants’ racial and ethnic information.

Only 3 studies indicated an intent to develop content and interventions optimized for use by people from marginalized populations (Multimedia Appendix 1). Two studies described developing interventions with input from African American youth or adolescents [35,37]. However, neither of these studies commented on how the content of their interventions was adapted to fit the needs of African Americans. One study was designed to be culturally relevant, appropriate, accessible, and engaging (ie, for Māori people, New Zealand’s indigenous population) [60].

### Addressing Sociocontextual Influences on Health

No studies reported that their intervention was designed to accommodate challenging sociocontextual factors, such has having too little income to be able to afford products or services designed to improve health (eg, gym membership, fresh fruits, and vegetables).

### Description of How TAMIs Affect Behavioral and Health Outcomes

Of the 34 studies, 30 were RCTs and reported outcomes, including alcohol consumption, tobacco use, hemoglobin A_1c_ levels, and human immunodeficiency virus viral load (see Multimedia Appendix 1 for a complete list). Of these 30 studies, 23 reported that the TAMI resulted in statistically significant improvements in the target behavioral and health outcomes [29,32,34,35,37-42,44-48,50,51,53,55-57,60,61] and the remaining 7 stated that the TAMI had no significant effect on behavior compared to the control group [28,30,31,43,49,52,54]. No RCTs reported that the TAMI resulted in worse outcomes compared to controls. The last 4 (out of 34) studies reporting behavioral and health outcomes used either a pretest-posttest design with no control group or a 1 group posttest only design [33,36,58,59]. All reported that the TAMI had a beneficial impact on promoting the desired health behavior. Eleven of the 34 studies examined feasibility, acceptability, or satisfaction, in addition to the behavioral or health outcomes [29,33,34,36,37,41,44,45,47,49,59]. All reported high participant ratings for these outcomes.

There were inconsistent reports about the value of adding CBT or other therapeutic components to an MI intervention. Some studies that reported combining MI and social determination theory (SDT) or MI and CBT did not achieve statistically significant increases in health behavior engagement compared to traditional web-based interventions [50,52,54]. One study reported that MI + CBT had a significant beneficial effect on alcohol consumption after 6 months [50]. Another study found that a web-based MI intervention with and without a trained psychologist coach was equally effective in decreasing the weekly standard units of alcohol at 6 weeks and 6 months compared to a wait-list control [56]. Two studies included in-person MI and reported a decrease in the number of drinking days in the TAMI group compared to the in-person MI and treatment as usual groups [44,48]. Finally, 1 study reported that adding an avatar to a web-based intervention did not significantly increase self-reported physical activity compared to the web-based intervention without an avatar [51], and the avatar did not create a stronger therapeutic relationship with participants.

Many studies noted that TAMIs could be useful in communities that have limited access to health care. Studies that focused on these communities or had many participants from these communities reported generally positive results, that is, the TAMI produced statistically significantly higher engagement in health promotion behaviors than the control treatment. Three studies designed specifically for underserved populations all reported that the TAMI intervention was more successful at promoting the desired target behavior than the control [35,37,60]. Specifically, 1 study noted that the intervention, which was designed specifically for the needs of Māori (New Zealand’s indigenous population) and non-Māori Pacific audiences, was equally effective for both populations and across different age groups [60]. Of the 18 studies reporting behavioral or health outcomes in a large proportion of participants who were racial or ethnic minorities or had limited incomes, 15 showed increases in engagement in healthy behaviors or better health outcomes [29,32-35,37-39,41,42,44-47,60]. Studies designed specifically for underinvested communities reported more success in promoting behavior change compared to studies that were not designed specifically for underinvested communities.

## Discussion

### Principal Findings

This scoping review extends prior work [23] by examining the following: (1) to what extent have individuals from marginalized sociodemographic groups been included in research on TAMIs; (2) how TAMIs offset sociocontextual influences on health, such as challenges imposed by the built environment or economics; and (3) how TAMIs may affect behavioral and health outcomes. The 34 studies reviewed here suggest that TAMIs likely improve health promotion and disease management behaviors, and health outcomes. However, the diversity in study designs, populations, and target behavioral or health outcomes preclude a formal meta-analysis at this point in time. The impact of TAMIs on health may be stronger among marginalized sociodemographic groups, including people from racial and ethnic minority backgrounds and those with low incomes. Although TAMIs have generally led to improvements in health promotion behaviors compared to control conditions, to date, TAMIs have been developed for a limited scope of health behaviors, the inclusion of individuals from marginalized sociodemographic groups has been minimal, and their impact on sociocontextual factors is not well understood.

Twenty-three studies reviewed reported that TAMIs were associated with statistically significant improvements in health promotion and disease management behaviors. However, 10 studies combined MI with other therapeutic approaches, like CBT, or intervention elements, such as counselor-mediated chat, that prevented an assessment of the unique impact of the TAMI. It is important to understand how these various therapeutic frameworks affected the results to identify what is necessary for a successful eHealth intervention. An experiment using the multiphase optimization strategy (MOST) design would allow investigators to parse which combination of several intervention components yields the greatest benefit for patients [64].

With the advent of more sophisticated technologies, such as machine learning and artificial intelligence, there have been many technological advancements in recent years, including the development of avatars capable of more personalized interactions and greater relational skills [24]. In fact, study participants who interact with a human-like virtual character may feel stronger social relations compared to interacting only with a plain text-based interface [65]. Yet, no study has specifically examined whether avatars improve an intervention’s target health behavior, despite calls [51] for future research to examine the effects of an avatar with more complex relational features. Such research may have implications for future uses of virtual reality, automated counseling or counselors, and other technological advancements for behavioral counseling, and offer a unique perspective on the importance of replicating human conversation in mHealth counseling technologies [66].

#### Scope of Health Behaviors and Outcomes Addressed by TAMIs

A majority of studies focused on substance, alcohol, or tobacco use (n=24), with the next most common behavioral targets being treatment or medication adherence (n=5) and diet or physical activity (n=2). This is consistent with the key health behaviors identified by the Centers for Disease Control and Prevention as being critical to promoting health and preventing chronic diseases [1]. However, there is potential to increase the scope of TAMI interventions. Health screenings can be critically important for detecting early disease in healthy populations and for preventing disease progression in populations with chronic health conditions [1,67]. For example, some studies screened for health metrics, such as BMI and hemoglobin A_1c_, which are very important for preventive screening [28,49]. In addition, health screenings are particularly important for high-risk populations, such as the nearly 17 million cancer survivors living in the United States [68], many of whom are vulnerable to experiencing the late effects of toxic treatments [69] but who may not be aware of the need to be screened. TAMIs that promote screening behaviors, particularly among cancer survivors, should be designed and tested. Such interventions may be particularly impactful for survivors of childhood cancers, who experience significant premature morbidity and mortality [70].

#### Inclusion of Individuals From Populations That Have Been Marginalized

Few of the studies reviewed were intentionally designed for marginalized sociodemographic groups. Although 59% (20/34) of the studies reviewed included an overrepresentation of individuals from marginalized groups, only 2 studies [37,60] explicitly stated that they received input from the marginalized communities for which the intervention was designed (ie, African American [37], and Māori and non-Māori Pacific individuals [60]). Only 2 studies included over 67% of individuals with low incomes [43,45]. However, no study addressed other factors, such as socioeconomic status, age, and sexual or gender identity. This is important because many of the studies reviewed suggested that TAMIs have the potential to be particularly beneficial for marginalized communities. For example, a study involving an MI intervention that was culturally tailored for Hispanic/Latino participants reported that “nearly all participants (95%) said that understanding their culture was important to understanding their drinking behavior” [71]. This suggests that TAMIs that consider the unique interests and needs of marginalized populations may have a more beneficial and sustainable impact than standard MI [20]. In addition, MI may be highly efficacious in minority populations due to its autonomy supportive approach rather than the authoritarian approach, which can trigger feelings of stigmatization and marginalization, that is commonly found in behavior change interventions [72,73]. However, research is needed to confirm this hypothesis. It also suggests that further studies that are more inclusive of marginalized participants are needed, so that the benefits of MI for groups that are at the greatest risk of experiencing health disparities can be better evaluated and, if appropriate, translated into practice and policy change.

#### Address Strategies for Overcoming Sociocontextual Barriers

In the studies we identified, there was scant attention to sociocontextual factors that shape and constrain people’s ability to engage in health promotion behaviors outside of race and ethnicity. This dearth of research is concerning, because it represents a missed opportunity to understand the role of TAMIs in overcoming powerful barriers to health behavior change [74]. For example, a TAMI focused on improving dietary behavior among people with low incomes might be more effective if information and other resources are provided to enable people living in food deserts to access fresh fruits and vegetables more easily. Similarly, a TAMI might offer information about strategies for exercising safely to people who indicate that they live in a neighborhood that is not conducive to outdoor exercise and cannot afford a gym membership.

### Comparison With Prior Work

A previous systematic review of TAMIs did not examine health outcomes or the potential relevance of TAMIs to people from marginalized or underserved sociodemographic groups [23]. Our research extends that work by evaluating these important characteristics. In addition, our findings indicating that TAMIs may be effective for improving health behaviors are consistent with the findings of previous reports [23].

### Limitations

This review should be considered in light of the following limitations. First, our analysis may have been limited by publication bias. While we found that a majority of studies reported positive results, those with negative results might remain disproportionately unpublished. The identified studies were also quite heterogeneous in populations, interventions, and outcomes assessed, restricting the conclusions that can be drawn when considering the results in aggregate. In addition, similar to a 2016 systematic review [23], only 1 study included a counselor-mediated MI group, so we are unable to report the efficacy of TAMIs compared to traditional MI interventions [23]. However, reports that TAMIs produce more beneficial changes in health promotion and disease management behaviors compared to no treatment or treatment as usual are critical, because traditional MI interventions are very costly and difficult to disseminate widely.

### Conclusions

MI has been largely successful in influencing positive behavioral change. Given the rapid increase in technological advancements in recent decades, TAMIs offer a low-cost and accessible platform to help patients improve their health. The results of this scoping review suggest that TAMIs are likely an effective way to promote positive behavioral change for the prevention and management of chronic diseases. TAMIs may hold particular promise for improving the health of marginalized communities, but few studies have described tailoring TAMIs in a culturally relevant, appropriate, or meaningful manner. In addition, no studies we reviewed addressed major sociocontextual factors that shape and constrain people’s ability to initiate and maintain changes in health behaviors. Studies that are more inclusive of communities that have been marginalized or underserved and that adequately address the sociocontextual factors shaping health could help reduce health disparities.

## Appendix

Multimedia Appendix 1 Characteristics of the studies included in the scoping review.

Multimedia Appendix 2 Additional characteristics of the studies included in the scoping review.

Multimedia Appendix 3 PRISMA (Preferred Reporting Items for Systematic Reviews and Meta-Analyses) checklist.

## Abbreviations

CBT: cognitive behavioral therapy
CIAS: Computerized Intervention Authorizing Software
MI: motivational interviewing
RCT: randomized controlled trial
TAMI: technology-delivered adaptation of motivational interviewing
